# A case of adrenocortical carcinoma accompanying secondary acute adrenal hypofunction postoperation

**DOI:** 10.1186/s12957-018-1326-5

**Published:** 2018-03-05

**Authors:** Kai Kou, Haiwen Zhang, Conggui Zhang, Enbo Xie, Yuguo Chen, Guangyi Wang, Guoyue Lv

**Affiliations:** Department of Hepatobiliary and Pancreatic Surgery, Bethune Hospital 1, Changchun, Jilin 130021 China

**Keywords:** Adrenocortical carcinoma, Diagnosis, Therapy, Immunohistochemistry, Mitotane

## Abstract

**Background:**

Adrenocortical carcinoma (ACC) is a rare, heterogeneous malignancy with a poor prognosis. ACCs are classified as functioning and non-functioning. The pathogenesis of ACC remains elusive, and diagnosis of ACC is currently based on pathology. In the absence of other effective approaches, surgical resection is the preferred treatment option.

**Case presentation:**

Here, we report a case of ACC in the retroperitoneum. The patient underwent radical adrenalectomy and remained disease-free throughout a 6-month follow-up.

**Conclusions:**

Radical surgical resection is an efficient therapy for ACC, and hydrocortisone can be used to alleviate symptoms of secondary acute adrenal hypofunction.

## Background

Adrenocortical carcinoma (ACC) is a rare, heterogeneous malignancy with a very poor prognosis. The overall incidence of ACC is approximately 0.5–2 cases per million per year [1], and it accounts for approximately 0.02–0.2% of all cancer-related deaths [2]. ACCs are classified as functional or nonfunctional based on the hormonal syndromes they produce. Functioning ACC usually manifests with hormonal syndromes, including virilization, Cushing’s syndrome, Con’s syndrome, and feminization. Nonfunctioning adrenal tumors remain a challenge in terms of early diagnosis and successful management, as there are no early signs or symptoms of disease [3].

Currently, the diagnosis of ACC is based on pathology and dependent on the Weiss score, tumor size, and underlying genetic predisposition [4]. Weissferdt et al. demonstrated positive staining for steroid receptor coactivator-1 (SRC-1) (39/40; 97.5%), inhibin-α (37/40; 92.5%), calretinin (32/40; 80%), synaptophysin (29/40; 72.5%), melan A (26/40; 65%), and cell adhesion molecule (CAM) 5.2 (9/40; 22.5%) in ACC [5]. Several genes and pathways with potential for use as diagnostic or prognostic markers have been identified using comparative genomic hybridization, DNA methylation profiling, and genome-wide mRNA and miRNA expression profiling [6].

Surgery is the mainstay treatment option for ACC. Chalasani et al. [7] reported that stage I and stage II ACC can be cured by surgical treatment, while an appropriate dose of oral mitotane may be beneficial for stage III and stage IV diseases.

Here, we report a case of a 31-year-old woman who was diagnosed with ACC. The patient was treated with left radical adrenalectomy and then followed up with physical assessments, laboratory testing, and imaging, including X-rays and computed tomography (CT) scans. At the 6-month follow-up, she remained disease-free.

## Case presentation

A 31-year-old woman presented with abdominal pain and fever for 4 days and unintended, excessive weight loss of 15 kg over the previous 2 months. Physical examination revealed vital signs within the normal range, except for intermittent fever reaching a maximum temperature of 39 °C once or twice a day. The patient had been ordinarily healthy, although she had a 15-year history of smoking. The patient had undergone two cesarean sections in January 2006 and June 2015 and had a 12-cm postoperative scar on the lower abdomen. In addition, she had a large palpable mass in the left abdomen.

Laboratory testing revealed a hemoglobin level of 112 g/L (normal, 115–150 g/L), indicating mild anemia. Coagulation parameters were: prothrombin time 14.8 s (normal, 9–13 s), international normalized ratio 1.26 (normal, 0.8–1.2 s), prothrombin activity 69 (normal, 80–120), and fibrinogen 7.65 g/L (normal, 2–4 g/L), suggesting coagulation was impaired. The tumor marker neuron-specific enolase was abnormally elevated at 35.84 ng/ml (normal, < 25 ng/ml). Liver function tests, urinalysis, serum electrolyte levels, and fasting blood glucose concentrations were normal.

**Fig. 1:**
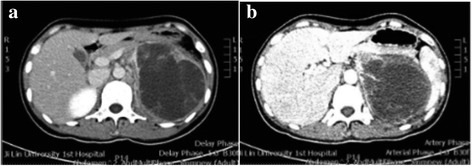
Computed tomography (CT) scans showing a mixed solid and cystic lesion (**a**) that was enhanced on contrast-enhanced CT (**b**)

**Fig. 2:**
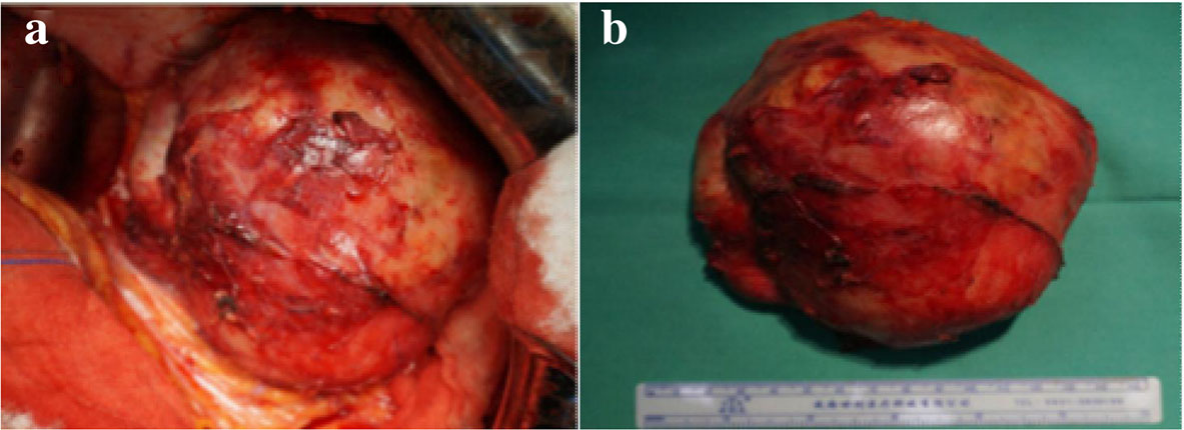
Intraoperative (**a**) and postoperative (**b**) photographs of the lesion

CT identified a mixed solid and cystic lesion with dimensions of 12.8 × 8.9 × 11 cm [Fig. 1] as well as dissepiment and calcification in the left upper quadrant of the abdomen. The left adrenal gland was not clearly visible. Multiple lymph nodes on the left side of the abdominal aorta were enlarged. The lesion and lymph nodes were enhanced on contrast-enhanced CT [Fig. 2].

**Fig. 3:**
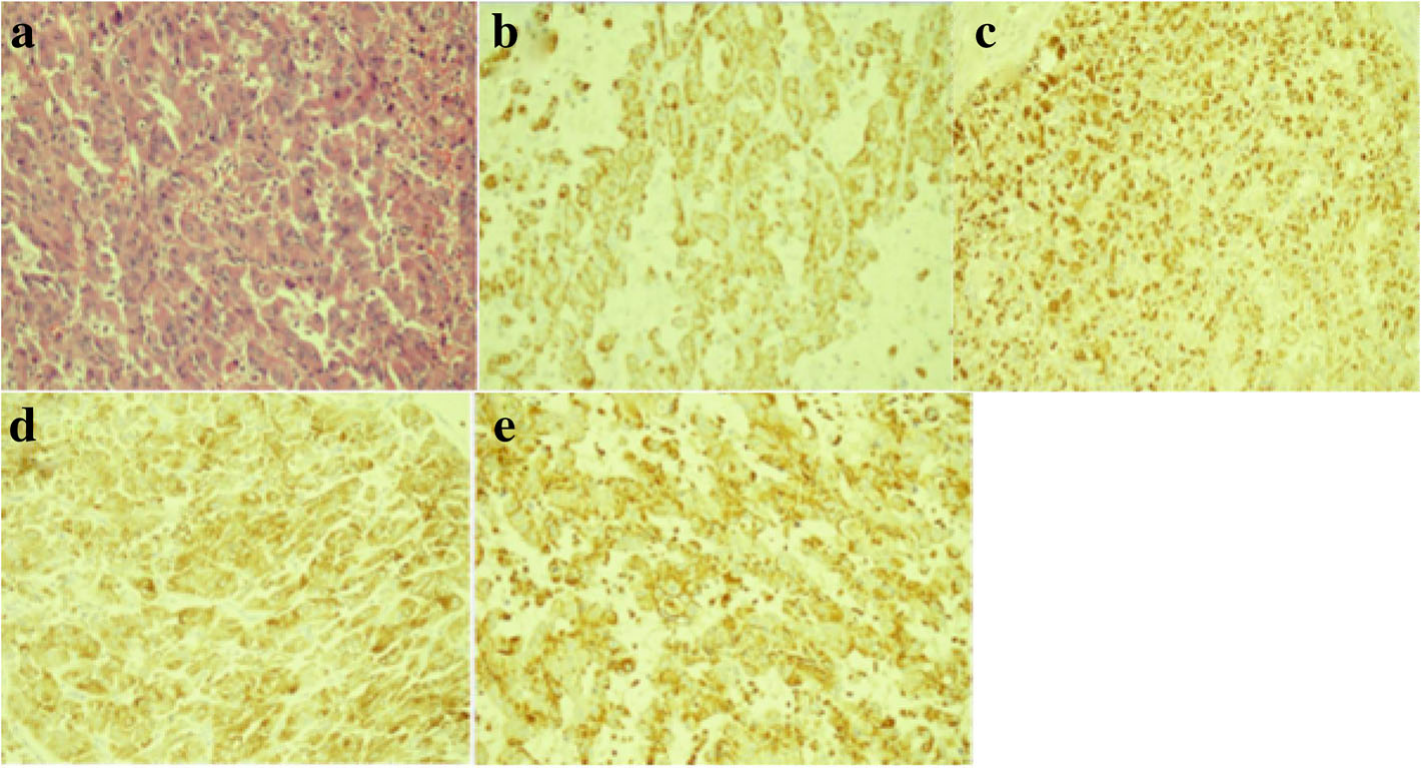
Histological staining of sections of resected tumor showing significant nuclear atypia, hyperchromasia, and pleomorphism of the tumor cells (**a**) and positive staining for CK-pan (**b**), vimentin (**c**), melan-A (**d**), and synaptophysin (**e**)

Radical surgical resection of the lesion was performed due to the patient’s severe pain and frequent fever. During the operation, the lesion and para-aortic lymph node were removed. After surgery, the patient presented with intermittent fever and rash. Pathological examination revealed an ACC tumor measuring 12 × 11 × 6 cm with extensive vessel invasion. The tumor was surrounded by a fibrous capsule and demonstrated no definitive evidence of capsular extension or invasion. No cancer was detected in the lymph nodes. Histological staining of sections of resected tumor certificated the diagnosis of ACC [Fig. 3]. The patient’s symptoms were assumed to be caused by secondary acute adrenal hypofunction. Subsequently, this was confirmed by measuring serum cortisol levels, which were 152.55 nmol/l at 00:00(normal, 240–619 nmol/l), 177.37 nmol/l at 08:00(normal, 240–619 nmol/l), and 139.1 nmol/l at 16:00 (normal, < 276 nmol/l). Upon treatment with hydrocortisone, the patient’s symptoms were resolved. The patient was discharged at 1 week postoperatively. The dose of hydrocortisone was gradually reduced and then discontinued at 1 month postoperatively. The patient underwent regular follow-up with physical assessments, laboratory testing, and imaging, including X-rays and computed CT scans. At the 6-month follow-up, she remained disease-free.

## Discussion

We report the case of a 31-year-old female who underwent radical surgical resection of a mixed solid and cystic lesion in the left upper quadrant of the abdomen. Postoperatively, the patient experienced intermittent fever and rash. Her symptoms resolved after hydrocortisone treatment, indicating that they were due to secondary acute adrenal hypofunction.

According to the 2004 World Health Organization (WHO) classification, ACC variants include oncocytic ACCs, myxoid ACCs, and ACCs with sarcomatous areas. Patients of all ages can present with ACC, but it has a bimodal age distribution with peaks in the first and fourth decades. Evidence suggests that approximately 60% of tumors are functional [8], and that patients may present with Cushing’s syndrome, virilization, feminization, or hypertension resulting from excessive secretion of glucocorticoids, androgens, estrogens, or aldosterone, respectively. Due to their silent nature, nonfunctioning tumors remain undiagnosed until late stage disease, when they mostly present as a large mass, as in the current case. Adrenocortical tumors are associated with weight loss, weakness, fever, and myalgias [8], as experienced by our patient, who also suffered from intermittent rash after surgery due to secondary acute adrenal hypofunction.

The pathogenesis of ACC remains elusive, but some molecular mechanisms have been implicated in its development, including pathways involving tumor protein 53 (TP53), insulin-like growth factor-2 (IGF-2), and β-catenin. In some studies, the presence of inactivating TP53 mutations and activating β-catenin mutations in ACC on transcriptome analysis was associated with worse prognosis. Several genome-wide expression studies demonstrated that IGF-2 can be used in combination with Ki-67, with 96% sensitivity and 100% specificity, in the diagnosis of ACC [6].

According to the TNM (tumor [T], lymph node [N], distant metastasis [M]) system of the American Joint Committee on Cancer, clinical staging of ACC includes: stage I, stage II, stage III, and stage IV [8]. In general, the larger the tumor, the greater the potential for malignancy [6]. Common metastatic sites include lung and liver, and less common sites are bone and bone marrow [9]. CT scanning plays an important role in defining the extent of the primary tumor as well as assessing the presence of metastatic disease.

Several clinical features are helpful in the diagnosis of ACC, including a high Weiss score, large tumor size, and underlying genetic predisposition, but definitive diagnosis depends on pathology. Metastasizing adrenocortical tumors are categorized by five histologic features, including capsular invasion, vascular invasion, diffuse tumor growth, spindle-cell morphology, and nuclear heterogeneity; malignant tumors have at least three of these features [5]. In the current case, a diagnosis of stage III ACC was reached based on the tumor size and enlargement of lymph nodes.

Surgical resection is the mainstay treatment for ACC [10]. Radical surgery represents nephrectomy, adrenalectomy, and resection of the surrounding lymph nodes. In some cases, multiple visceral resections are required to achieve a complete R0 resection.

Bacalbasa et al. [11] reported the case of a 65-year-old patient treated with a complete resection en bloc with left nephrectomy and adrenalectomy, distal pancreatectomy, splenectomy, left colectomy, and para-aortic lymph node dissection. In the modern pursuit of minimally invasive technology with respect to aesthetics, posterior retroperitonoscopic adrenalectomy (PRA) has become a standard approach for removal of the adrenal gland [12].

In recent years, mitotane has been advocated as a therapy following the resection of a localized tumor [13] and for metastatic disease. Sabolch et al. [14] showed that radiotherapy significantly lowered the risk of local recurrence/progression in patients with ACC. Increasing our understanding of the pathogenesis of ACC may provide potential treatment targets, including 9-cisRA [15], miRNAs [14], and topoisomerase 2-alpha (TOP2A) [16].

## Conclusions

ACC has a poor prognosis with frequent recurrence and metastases even after complete resection. The median survival times for patients with stage I–II, stage III, and stage IV diseases are 159 months (95% confidence interval [CI], 93–225 months), 26 months (95% CI, 4–48 months), and 5 months (95% CI, 2–7 months), respectively [17]. Our patient was still disease-free at 6 months after tumor resection.
